# Plasmids for Independently Tunable, Low-Noise Expression of Two Genes

**DOI:** 10.1128/mSphere.00340-19

**Published:** 2019-05-29

**Authors:** João P. N. Silva, Soraia Vidigal Lopes, Diogo J. Grilo, Zach Hensel

**Affiliations:** a Instituto de Tecnologia Química e Biológica António Xavier, Universidade Nova de Lisboa, Oeiras, Portugal; University of Iowa

**Keywords:** expression systems, flow cytometry, fluorescent-image analysis, heterologous gene expression, recombinant-protein production, regulation of gene expression, transcriptional regulation

## Abstract

Some microbiology experiments and biotechnology applications can be improved if it is possible to tune the expression of two different genes at the same time with cell-to-cell variation at or below the level of genes constitutively expressed from the chromosome (the “extrinsic noise limit”). This was recently achieved for a single gene by exploiting negative autoregulation by the tetracycline repressor (TetR) and bicistronic gene expression to reduce gene expression noise. We report new plasmids that use the same principles to achieve simultaneous, low-noise expression for two genes in Escherichia coli. The TetR system was moved to a compatible plasmid backbone, and a system based on the *lac* repressor (LacI) was found to also exhibit gene expression noise below the extrinsic noise limit. We characterized gene expression mean and noise across the range of induction levels for these plasmids, applied the LacI system to tune expression for single-molecule mRNA detection under two different growth conditions, and showed that two plasmids can be cotransformed to independently tune expression of two different genes.

**IMPORTANCE** Microbiologists often express foreign proteins in bacteria in order study them or to use bacteria as a microbial factory. Usually, this requires controlling the number of foreign proteins expressed in each cell, but for many common protein expression systems, it is difficult to “tune” protein expression without large cell-to-cell variation in expression levels (called “noise” in protein expression). This work describes two protein expression systems that can be combined in the same cell, with tunable expression levels and very low protein expression noise. One new system was used to detect single mRNA molecules by fluorescence microscopy, and the two systems were shown to be independent of each other. These protein expression systems may be useful in any experiment or biotechnology application that can be improved with low protein expression noise.

## Abstract (precis)

Microbiologists often express foreign proteins in bacteria in order study them or to use bacteria as a microbial factory. Usually, this requires controlling the number of foreign proteins expressed in each cell, but for many common protein expression systems, it is difficult to “tune” protein expression without large cell-to-cell variation in expression levels (called “noise” in protein expression). This work describes two protein expression systems that can be combined in the same cell, with tunable expression levels and very low protein expression noise. One new system was used to detect single mRNA molecules by fluorescence microscopy, and the two systems were shown to be independent of each other. These protein expression systems may be useful in any experiment or biotechnology application that can be improved with low protein expression noise.

## INTRODUCTION

We recently reported the development of a plasmid-based gene expression system in which a gene of interest was expressed bicistronically with the tetracycline repressor (TetR) (1). Using this gene expression system, cell-to-cell variation was below the “extrinsic noise limit” (coefficient of variation squared of protein concentration, CV^2^, ≈0.1) observed for genes expressed from the chromosome (2). When TetR and green fluorescent protein (GFP) were expressed bicistronically, GFP induction and gene expression noise were similar to those observed for a TetR-GFP fusion protein with autoregulation (3). Compared to induction of gene expression under the control of a constitutively expressed transcriptional repressor, the inducer dose response was relatively linearized, and gene expression noise was much lower at intermediate induction levels (1).

Our experiments in mRNA detection and other single-molecule applications in living Escherichia coli cells sometimes require the tunable expression of two different genes, both with low noise levels. For example, adopting a recently reported mRNA detection system based on local enrichment of fluorescent RNA-binding proteins (4) for use in E. coli requires lower noise in protein production than in the same system in Saccharomyces cerevisiae because of a smaller cell volume for E. coli and the inability to sequester unbound protein in the nucleus. At the same time, tunable expression with low noise in the level of the target RNA is desired to make it possible to characterize the accuracy of RNA detection over a range of RNA levels. We hoped that expressing both the target RNA and RNA-binding fluorescent protein on two plasmids that could be tuned independently would simplify and accelerate the development of new RNA detection systems in E. coli. Achieving this was a three-step process: first, characterizing the TetR-based system on a compatible plasmid backbone; second, establishing an orthogonal, low-noise expression system based on the *lac* repressor (LacI); and third, showing that the two systems can be tuned independently.

## RESULTS

### Moving a bicistronic autoregulatory construct to a compatible plasmid backbone.

The first step in creating a low-noise system for tuning the expression of two genes was to establish that a previously characterized, bicistronic autoregulatory circuit functions well in a compatible plasmid backbone. In this expression system, GFP and TetR are expressed bicistronically from the TetR-repressible promoter P_LtetO-1_ and expression is induced by the addition of anhydrotetracycline (ATc) (1). This system was shown to have low noise and a linearized dose response compared to those of a system in which TetR was constitutively expressed. We moved the system from an ampicillin resistance-conferring plasmid with a p15A origin of replication to a lower-copy-number plasmid with a pSC101 origin and spectinomycin resistance (5). The p15A and pSC101 origins have been used together in multiplasmid systems (6).

**FIG 1:**
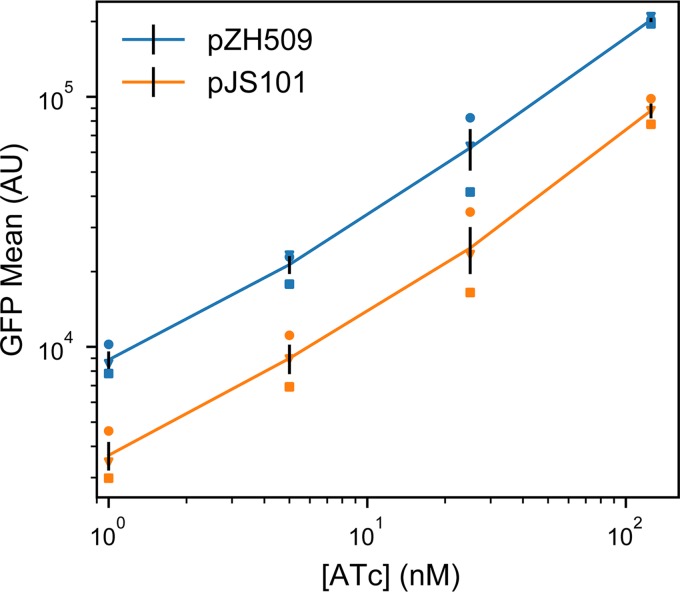
Moving the TetR expression system to a compatible plasmid backbone. Cultures of E. coli MG1655 harboring pZH509 (p15a origin) or pJS101 (pSC101 origin) expressing GFPmut2 with bicistronic autoregulation by TetR were grown at 30°C in rich medium with induction by 1, 5, 25, and 125 nM ATc. Mean single-cell GFP fluorescence was estimated using flow cytometry. Mean GFP levels in 3 independent replicates are indicated with different shapes. Black lines indicate the mean plus or minus 1 standard error of the mean from the 3 replicates.

The GFP expression mean and noise were characterized from low to high levels of induction by flow cytometry. Figure 1 shows that pJS101 induced expression at ATc concentrations similar to those of pZH509; the change to the lower-copy-number pSC101 backbone resulted in a 58% drop in mean expression levels at a wide range of ATc concentrations. For a similar expression system in the absence of autoregulated TetR expression, moving the P_LtetO-1_ promoter from a p15A to a pSC101 backbone resulted in an 87% drop in expression (7). A smaller change was expected in our experiment, since negative autoregulation provided dosage compensation, just as autoregulation can reduce noise in plasmid copy number (3, 8, 9).

### Alternative regulatory construct with LacI replacing TetR.

We hypothesized that replacing P_LtetO-1_ with the isopropyl-β-d-thiogalactopyranoside (IPTG)-inducible promoter P_LlacO-1_, which has similar characteristics (7), and replacing TetR with LacI might result in a similarly useful expression system that could be tuned independently. However, regulatory parameters for TetR and LacI vary significantly. TetR binds *tetO2* more strongly than LacI binds *lacO1* (there is an approximately 0.5 to 1.0 kcal/mol difference in binding energy [10, 11] for a single site, with 2 tandem sites in our constructs). Further, TetR binds ATc much more strongly than LacI binds IPTG (the magnitude of difference in typical concentrations required for half-induction is over 3 orders of magnitude [12, 13]).

**FIG 2:**
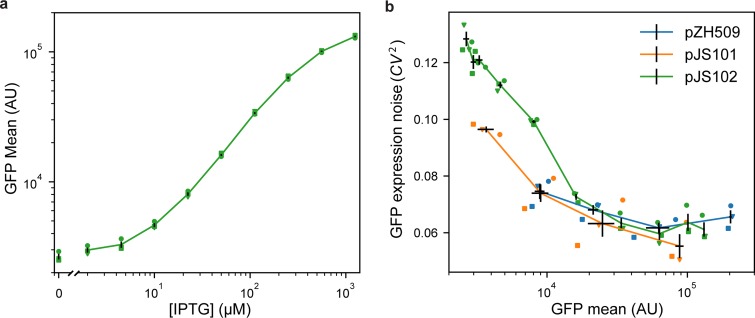
Characterizing mean expression levels and noise for different gene expression systems. Cultures of E. coli MG1655 harboring pJS102 (LacI regulation, p15a origin, green), pZH509 (TetR regulation, p15a origin, blue), or pJS101 (TetR regulation, pSC101 origin, orange) were grown at 30°C in rich medium with induction by IPTG (0, 2, 4.5, 10, 22.4, 50, 111.8, 250, 559, and 1,250 μM) or ATc (1, 5, 25, and 125 nM). Mean single-cell GFP fluorescence and GFP expression noise were estimated using flow cytometry. Data from 3 independent replicates are indicated with different shapes. Black lines indicate the mean GFP expression and expression noise ± 1 standard error of the mean from the 3 replicates. (a) Mean GFP expression for pJS102 at different IPTG concentrations shows tunable and reproducible induction over an ∼50-fold dynamic range. Expression at zero IPTG is plotted separately to fit on a logarithmic scale. (b) GFP expression noise as a function of the mean for pZH509, pJS101, and pJS102. For all samples, the GFP mean increases monotonically with inducer concentration. Note that the full range of induction is not shown here for pZH509 and pJS101 as even 1 nM ATc induces expression. GFP expression noise is low under all conditions for all strains. AU, arbitrary units.

We first characterized induction of GFP expression in MG1655 cells harboring IPTG-inducible pJS102 by flow cytometry. Figure 2a shows an induction range of almost 2 orders of magnitude, from 0 to 1,250 μM IPTG, with very good reproducibility of induction levels in 3 independent experiments. Previous experiments with the TetR-based system showed a similar total dynamic range but with a large jump in expression going from 0 nM to 0.5 nM ATc (1). This effect is not seen for pJS102, suggesting that switching from TetR-ATc to LacI-IPTG improves the dynamic range of tunable induction levels to a small extent.

Next, we compared levels of noise in protein expression, with the concern that the *lac* operon present in the MG1655 host strain might lead to all-or-no expression at intermediate IPTG concentrations (14). However, Fig. 2b shows low noise in GFP expression at all IPTG concentrations, with noise levels comparable to those of pZH509 and pJS102 at the same mean GFP levels. Note that, apparently, high noise at very low expression was partially due to measurement noise and, at any rate, was much lower than noise when expression was regulated by a constitutively expressed repressor (1).

We found that side scattering was weakly correlated with fluorescence and, thus, with cell size, so gating for scattering modestly reduced measured noise in fluorescence intensity. However, we compared this noise to an “extrinsic noise limit” determined from measurements of cell fluorescence divided by cell area (2), which effectively does the same thing. In practice, we observed slightly lower noise measurements for GFP concentrations in fluorescence microscopy images than for total GFP fluorescence in the gated flow cytometry sample for similarly induced strains. This difference was more significant for very low-expression conditions, and noise under conditions where GFP fluorescence distributions significantly overlapped ungated background events (GFP intensity of less than 10^4^) was somewhat overestimated. Our noise measurements were also consistent with the lower limit of gene expression noise found in many E. coli promoters using a similar flow cytometry method with similar gating and fitting procedures (15).

### Using the new induction system for detection of single mRNA in living E. coli cells.

Recently, an improved method for detection of mRNA by local enrichment of fluorescent RNA-binding proteins was reported for S. cerevisiae (4). This reduced the aggregation of mRNAs bound by the bacteriophage MS2 coat protein, which has also caused mRNA immortalization that has limited experiments in E. coli to observing transcription just after induction (16). We hypothesized that aggregation may be reduced by reducing the expression levels of both mRNA and mRNA-binding proteins and by having low cell-to-cell variation in expression. We developed a strain in which mRNA molecules encode mVenus-Cro and include 24 tandem repeats of the binding sequence for the PP7 coat protein (PP7cp) (17). These mRNAs were constitutively expressed at low levels (less than 1 molecule per cell). Plasmid pJS102 was used as a template to develop a fluorescent, IPTG-inducible reporter of expression, PP7cp-SYFP2.

**FIG 3:**
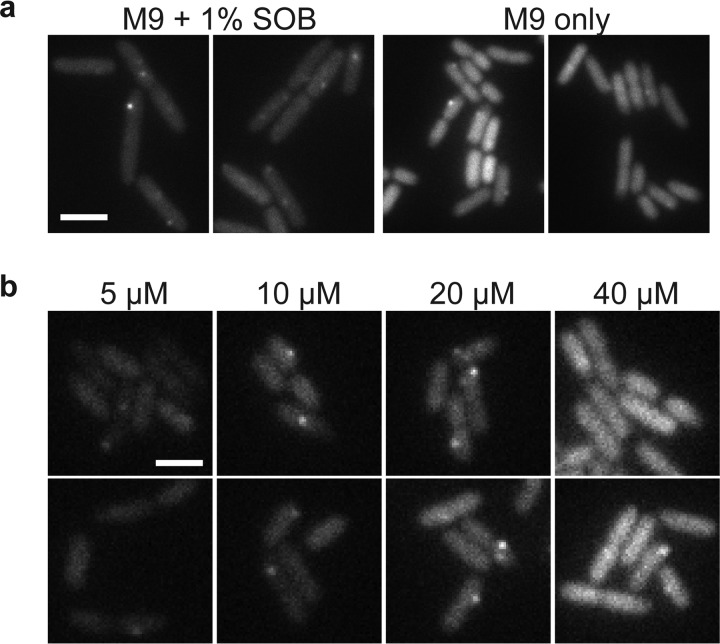
Use of IPTG to tune the expression of a fluorescent RNA-binding protein for single mRNA detection under different growth conditions. ZHX99 cells were grown with low expression levels (less than 1 molecule per cell) of mRNAs harboring 24 tandem binding sites for the PP7 coat protein fused to SYFP2. Cells were spotted on agarose gel pads, and yellow fluorescent protein (YFP) fluorescence images were acquired. Two sample images are shown for each condition. Images were taken shortly after samples were prepared, so adjacent cells were not usually closely related in cell lineages. (a) PP7cp-SYFP2 was induced with 100 μM IPTG to detect single mVenus-Cro mRNA molecules under supplemented-medium and minimal-medium conditions; in minimal medium, there was too high a PP7cp-SYFP expression level to see single mRNA spots above background. Scale bar, 4 μm. (b) With the pJS102 expression system, PP7cp-SYFP2 expression levels were varied by induction with 5, 10, 20, and 40 μM IPTG. The range of 10 to 20 μM IPTG was identified to give bright mRNA spots above the background of unbound PP7cp-SYFP2 molecules. Scale bar, 2 μm.

We tested the utility of this expression system for tuning low-noise gene expression under different growth conditions. In previous experiments, we expressed the RNA-binding protein from a constitutive promoter integrated into the chromosome; this required long cycles of optimization every time a parameter was changed (e.g., growth medium, temperature, and fluorescent protein sequence) that changed protein expression levels. Figure 3a shows that single-molecule mRNA detection was optimal (fluorescent mRNA spots are sufficiently bright but not obscured by background) at 100 μM IPTG in minimal medium supplemented with 1% rich medium. We note the absence of pole-localized fluorescent spots that characterize mRNA aggregation (18), and we observed reasonable mRNA lifetimes of a few minutes in time-lapse imaging.

We moved to minimal media to explore a growth condition with different mRNA expression levels and lower growth rates but found that 100 μM IPTG gave a background of unbound PP7cp-SYFP2 molecules that often made it impossible to detect mRNA spots. Figure 3b shows how the IPTG-inducible expression system made it simple to quickly scan different PP7cp-SYFP2 induction conditions and identify 10 to 20 μM IPTG as a range in which PP7cp-SYFP2 levels were high enough to label single mRNAs but not so high as to give a high background of unbound molecules. Lastly, we note that the strain used for mRNA imaging has its entire *lac* operon replaced by the synthetic construct. Thus, this expression system works well both in the presence and in the absence of the *lac* operon.

### Independent, tunable expression of two genes.

**FIG 4:**
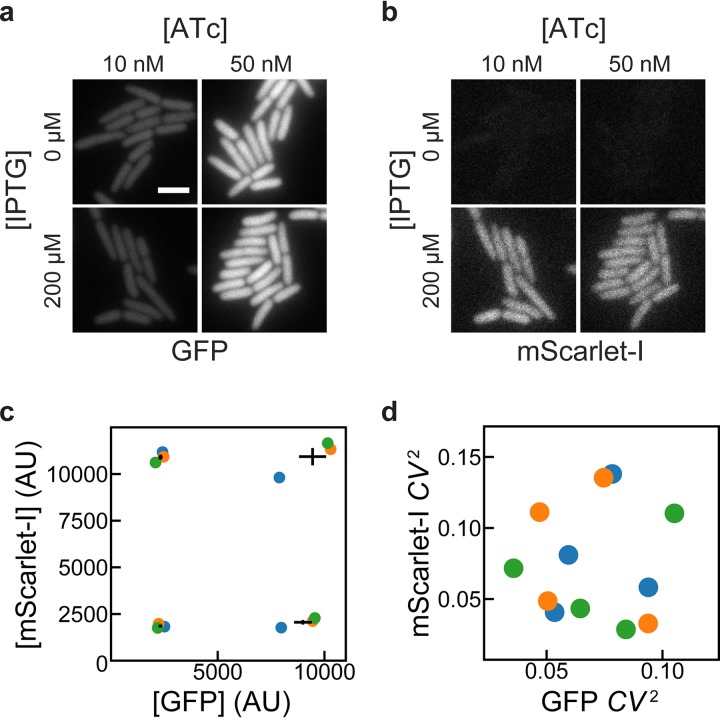
Independence of induction of TetR and LacI systems. MG1655 cells harboring pJS101 (ATc-inducible expression of GFP, pSC101 origin) and pDG101 (IPTG-inducible expression of mScarlet-I, p15a origin) were grown with different combinations of IPTG and ATc concentrations. GFP and mScarlet-I fluorescence was observed and quantified by fluorescence microscopy. (a) GFP fluorescence showed no apparent influence of IPTG on ATc-induced expression of GFP for cells grown at 30°C in EZ-Rich medium. (b) For the same cells as in panel a, no influence of ATc was observed on IPTG-induced expression of mScarlet-I. Scale bar, 3 μm. (c, d) Cells were grown at 37°C in M9A medium in 3 independent replicates, and the mean and noise of GFP and mScarlet-I fluorescence intensities (from the average intensities of pixels within cells) were estimated at different IPTG (50, 1,250 μM) and ATc (5, 125 nM) concentrations. Translation was inhibited with chloramphenicol for 1 h prior to the preparation of microscope samples to allow for fluorescent-protein maturation. Colored circles indicate the results from independent replicates. For mean expression levels in panel c, black lines indicate the mean GFP and mScarlet-I expression ± 1 standard error of the mean from the 3 replicates. Noise measurements in panel d indicate low noise in both channels under all conditions and in all replicates.

Lastly, we tested whether ATc-inducible and IPTG-inducible plasmids could be combined to achieve low-noise expression of two genes in the same cell. We replaced GFPmut2 in pJS102 with the fast-maturing red fluorescent protein (RFP) mScarlet-I (19) to create the plasmid pDG101. This plasmid was cotransformed with pJS101 into E. coli MG1655, and levels of green and red fluorescence were compared at different combinations of ATc and IPTG concentrations. Figure 4a shows that pJS101 induction by ATc was unaffected by pDG101 induction by IPTG and that all conditions gave low noise in GFP concentration. Figure 4b shows that mScarlet-I expression from pDG101 was similarly unaffected by the level of pJS101 induction by ATc. Figure 4c and d show that mean expression levels were reproducible in 3 independent replicates, with low noise in each sample across a 5-fold increase in expression levels. There is some day-to-day variation in mean expression levels, but for each replicate, inductions of GFPmut2 and mScarlet-I are independent. Thus, independent, tunable expression of two genes can be achieved by replacing GFPmut2 in pJS101 and pJS102 with other genes of interest and cotransforming the plasmids into E. coli.

## DISCUSSION

### Implementing low-noise expression systems.

Using modern molecular cloning techniques, it is simple to replace GFPmut2 in pJS101 and pJS102 with genes of interest by PCR and isothermal assembly, with near 100% efficiency and a low probability of clones with incorrect sequences. The apparent insensitivity of this circuit to regulatory parameters, such as binding affinities for the repressor to DNA and inducer, suggests that it can be easily extended to a third, repressor-based expression system. Further, additional ribosome binding sites can be added to the bicistronic operon to express additional components. Notably, we have found that both the ATc- and IPTG-inducible systems function well in MG1655, a strain in which the *lac* operon was deleted, and in the E. coli TOP10 strain. This host insensitivity may be specific to repressors that are not encoded by the host strain, such as TetR, or repressors that are expressed at very low levels, such as LacI (20).

### Functionality in other organisms.

We chose p15a and pSC101 plasmids for these systems because we apply them primarily in E. coli, they transform efficiently, and they have been successfully cotransformed in earlier work (6). However, these are narrow-host-range plasmids, and it remains to be seen whether our expression systems will work well in broad-host-range plasmids (21), in other organisms, or upon chromosome integration. We expect that the system will be reasonably portable in hosts meeting some basic criteria. First, the P_LtetO-1_ and P_LlacO-1_ promoters are very strong, with sequences close to the E. coli σ^70^ consensus; this promoter must match promoter sequences recognized in another system. Second, we have created expression systems using a variety of ribosome binding sites with different translation rates (1); translation rates can be predicted from homology to 16S rRNA and other factors independent of the host (22) and may need to be modified to achieve a desired range of induction. Third, we used the strong, Rho-independent rrnB T1 terminator (23), which should work in a broad range of microbial hosts but may be less effective in some. Lastly, the addition of an insulating transcriptional repressor ahead of the P_LtetO-1_ and P_LlacO-1_ promoters is likely to reduce sensitivity to transcription upstream of these constructs. We also note that noise for pJS101, with its low-copy-number pSC101 replicon, is lower than that for pZH509 or pJS102 at similar expression levels (Fig. 2b). This suggests that incorporating this construct into the chromosome, where copy number is more tightly regulated, may lead to further noise reduction.

### Possible applications.

We expect that the expression plasmids introduced here will be useful for diverse applications in molecular biology. Expression and purification of heteromeric protein complexes may be improved by stoichiometric production of their components, mimicking proportional synthesis in natural systems (24). Additionally, low-noise expression can improve protein production yields (25). These systems may also be used in synthetic biology applications where yields can be improved by sequential induction of different components with low cell-to-cell variability. The capacity for low-noise expression at very low expression levels makes them particularly promising for single-molecule imaging experiments or for recombinant expression of low-copy-number components with low cell-to-cell variation to reproduce chromosomal expression levels. We see two major drawbacks to our gene expression system. First, the dynamic range of inducible expression is lower than for systems controlled by constitutively expressed transcriptional repressors (1, 7), because some expression must occur at a zero inducer concentration before negative feedback kicks in. While the dynamic range might be expanded by increasing repressor binding strength or having a low level of constitutive repressor expression, we have yet to succeed in this. Second, TetR and LacI are expressed at different levels under different induction conditions. This may have off-target effects (e.g., from nonspecific DNA binding); we have occasionally observed slow growth at very high induction levels (over 200 nM ATc for pZH509), but we have not tested whether this is due to high TetR expression or high GFPmut2 expression.

## MATERIALS AND METHODS

### Strain construction.

All plasmids were constructed using isothermal assembly (26) of fragments generated by PCR or double-stranded DNA synthesis (IDT, Coralville, IA) and transformed into Top10 E. coli cells (5-1600-020; IBA Life Sciences, Göttingen). Transformants were screened by colony PCR and verified by sequencing (Stab Vida, Caparica, Portugal). Purified plasmids were transformed into E. coli strain MG1655 by growing 3 ml of culture in superoptimal broth (SOB) medium at 30°C to an optical density at 600 nm (OD_600_) of 0.4, washing cells twice with 1 ml ice-cold water, resuspending them in 40 μl water, electroporating 1 to 10 ng plasmid with the EC1 setting of a MicroPulser (Bio-Rad Laboratories, Hercules), and allowing cells to recover for 1 h at 37°C in SOC medium (SOB with catabolite repression) before plating them on selective LB agar.

To generate pJS101 with a compatible backbone, plasmid pZH509 (1) was used as a template to amplify the bicistronic regulatory construct including the P_LtetO-1_ promoter (7), GFPmut2 (27), Tn*10* TetR (28), and the *rrnB* T1 transcription terminator (23). Using isothermal assembly, this construct was inserted into the pGB2 backbone (5) with the pSC101 origin of replication and spectinomycin resistance to generate plasmid pJS101. Plasmid pJS102 was generated by 3-fragment isothermal assembly. Plasmid pZH509 was used as a template both for the vector backbone and for GFPmut2, with nonhomologous extensions added to PCR primers to generate the P_LlacO-1_ promoter (7). LacI (20) was amplified from E. coli MG1655 by colony PCR.

The test strain for mRNA imaging, ZHX99, was constructed similarly to ZHX222 in recent work (29). In ZHX99, a construct in which a fusion protein of mVenus and Cro is expressed from the bacteriophage λ promoter *P_R_* was integrated into the chromosome to replace the *lac* operon in MG1655 (30). ZHX99 differs from ZHX222 in three ways. First, the *P_R_* promoter was weakened by site-directed mutagenesis to produce a strain with lower mRNA levels. Second, a very strong ribosome binding site (RBS) was added (RBS 136 [22]). Third, 24 tandem repeats of the recognition sequence for the PP7 coat protein (PP7cp) were inserted between the open reading frame and transcription terminator (amplified by PCR from pDZ251 [17]). The pZH713 plasmid for mRNA detection was constructed by replacing GFPmut2 in pJS102 with a fusion protein of SYFP2 (amplified from a plasmid [31]) and PP7cp (generated after codon optimization by DNA synthesis based on previously reported sequences [32]). Additionally, in pZH713, the PP7cp-SYFP2 fusion protein is translated from the weak ribosome binding site from pZH511 (1). We note that mVenus expression in ZHX99 is extremely low (undetectable without strong laser excitation) and does not interfere with mRNA detection by localizing up to 48 SYFP2 molecules in a diffraction-limited spot bound to a single mRNA molecule.

To test independent induction of two genes, GFPmut2 in pJS102 was replaced by mScarlet-I (amplified from a plasmid [19]) to make pDG101. Plasmids were cotransformed into MG1655 by electroporation according to the above protocol, except with 1 μl (each) undiluted plasmid (20 to 40 ng) and selection on LB agar plates with both spectinomycin and carbenicillin. Sequence maps are included in an online repository (33), and plasmids useful for constructing additional two-gene expression systems (pJS101 and pJS102) (Table 1) are available from Addgene (deposit no. 118280 and 118281) and have been verified by whole-plasmid sequencing (34).

**TABLE 1:** Plasmids used in this study^*a*^

Plasmid	Ori	GOI	Promoter	Reference
pZH501	p15a	CI-SNAP-tag	P_LtetO-1_	1
pZH509	p15a	GFPmut2	P_LtetO-1_	1
pJS101	pSC101	GFPmut2	P_LtetO-1_	This work
pJS102	p15a	GFPmut2	P_LlacO-1_	This work
pZH713	p15a	PP7cp-SYFP2	P_LlacO-1_	This work
pDG101	p15a	mScarlet-I	P_LlacO-1_	This work

a Ori is the origin of replication. GOI is the gene of interest, which in all plasmids is expressed from a bicistronic mRNA with the appropriate repressor (TetR or LacI). Plasmid pZH713 contains a weaker ribosome binding site than the other plasmids. Plasmid pJS101 confers spectinomycin resistance, and other plasmids confer ampicillin resistance.

### Characterization of GFP expression by flow cytometry.

All flow cytometry experiments were repeated 3 times on different days and used plasmids transformed by electroporation into E. coli MG1655. Cultures were grown overnight at 30°C from LB agar plates supplemented with carbenicillin or spectinomycin (both at 50 μg/ml) in 1 ml EZ Rich defined medium (M2105; Teknova, Hollister, CA) supplemented with the same antibiotics. Cells were reinoculated at 1:400 in 1 ml of the same medium supplemented with isopropyl-β-d-thiogalactopyranoside (IPTG) at concentrations of 0, 2, 4.5, 10, 22.4, 50, 111.8, 250, 559, and 1,250 μM or anhydrotetracycline (ATc) at concentrations of 1, 5, 25, and 125 nM, as indicated in the figures, and grown at 30°C for 4 to 4.5 h until they reached an OD_600_ of 0.2 to 0.3. ATc (Alfa Aesar catalog no. J66688) is an analog of tetracycline that binds the tetracycline repressor very strongly, allowing it to be used as an inducer in strains without tetracycline resistance (35). Next, 10 μl of cells was added to 1 ml of phosphate-buffered saline (PBS) at pH 7.4 and examined by flow cytometry.

Flow cytometry data were collected on an S3e cell sorter (Bio-Rad, Hercules, CA) using a target flow rate of 2,000 cps and collecting 30,000 counts for each sample. A 488-nm laser line was used for excitation at its maximum power setting, with amplification settings of 450 (forward scattering [FSC]), 350 (side scattering [SSC]), and 900 (FL1, 525/30 nm). The cell sorter is calibrated daily for a linear response to sample fluorescence intensity. Acquisition was triggered by forward scattering with a threshold of 3. Data were exported as an FCS file and imported into a custom Python script using FlowCal (36). Following previous methods (1), one-third of samples were selected based on proximity to the peak of FSC area and SSC height in a two-dimensional (2D) histogram using the density2d method in FlowCal. The FL1 area measurements were used to estimate the mean and variance of GFP distributions for all samples. This was done by estimating the probability density functions in bins distributed equally in logarithmic space and fitting by least-squares minimization to a gamma function. We found that this method reduced the influence of low-FL1 area events that escaped other gating steps and which had frequencies that varied for different samples and days (data and figures are available in an online repository [33]). In all plots, the mean fluorescence of a strain harboring a similar plasmid, pZH501, that does not encode a fluorescent protein was subtracted (1).

Noise was calculated as the coefficient of variation squared (CV^2^) from the mean, μ, and variance, σ^2^, as σ^2^/μ^2^. We chose CV^2^ to facilitate comparison with earlier work (1, 2). We note that gating by FSC area and SSC height to some extent selects for cells near the median cell size, so ignoring other sources of experimental error, we expect our noise measurements to fall somewhere between the noise in the number of proteins per cell and the noise in the protein concentration. In earlier work identifying the extrinsic noise limit, noise was estimated from integrated fluorescence intensities normalized by cell size (proportional to protein concentration) in microscope images (1). The script for data analysis as well as all raw flow cytometry data is available in an online repository (33) and utilized modules from SciPy, NumPy, Matplotlib, and Pandas.

### Microscopy.

All imaging was done on a Leica DMI6000 inverted microscope using illumination from a Leica EL6000 source (at various intensities, ensuring minimal photobleaching during acquisition), fluorescence filter cubes (Leica GFP ET, a custom filter set with Semrock filters FF01-561/13, FF02-616/73, and DI02-R561, or the Semrock LF514-B filter set), a 100×/1.46 a-plan apochromat oil immersion objective, Leica type F immersion oil, and an Evolve 512 electron microscopy charge-coupled device (EM-CCD) camera (Photometrics) using 16-bit EM gain amplification. Images were prepared using Fiji (37), with linear scaling and maintenance of minimum and maximum intensity values for all comparable images.

For mRNA imaging, overnight cultures were diluted 1:100 and grown at 30°C in M9 medium supplemented with 1× minimal essential medium (MEM) amino acids (M9A) or M9A medium additionally supplemented with 1% SOB medium for 2 to 4 h. Supplementation with SOB is used to provide quasi-rich growth conditions with very low fluorescence background and autofluorescence without the expense of commercial rich minimal media. We previously used this growth condition to characterize gene expression noise for different systems (1). Agarose gel pads (3% BP165-25; Fisher Bio-Reagents) were prepared with M9A medium with and without supplementation with 1% SOB, and the microscope sample chamber was maintained at 30°C.

To quantify independent induction from 2 plasmids, cells were grown in M9A medium at 37°C. Overnight cultures were diluted 1:100 in M9A medium supplemented with ATc and IPTG and grown for 3.5 h. At this point, chloramphenicol was added to a final concentration of 100 μg/ml, and cultures were incubated for an additional 1 h at 37°C to allow most GFPmut2 and mScarlet-I molecules to mature (maturation times of 5.6 and 25.7 min, respectively [38]). Cells were spotted onto agarose gel pads prepared with PBS and imaged at room temperature. For each of 3 replicates and 4 induction conditions, 10 images each were acquired in the bright-field, GFPmut2, and mScarlet-I channels. For analysis, all cells in these images were manually segmented using the selection brush tool in Fiji with a width of 10 pixels (163 to 212 cells per sample). This selection was used to extract the mean green and red intensities (proportional to the concentration of GFPmut2 and mScarlet-I molecules in the cell, respectively). For each image, the mean background intensity was also measured from a large region containing no cells, which was subtracted from each single-cell data point. Mean, variance, and CV^2^ were estimated from this data for each sample by following the same fitting protocol used for flow cytometry data. Sample images were acquired similarly, with growth instead in EZ-Rich medium at 30°C, supplemented with ATc and IPTG.

### Availability of data.

The raw data, single-cell intensities, Fiji macro, and Python scripts required to reproduce this analysis are available in an online repository (33).
